# On the use of stable oxygen isotope (*δ*^18^O) measurements for tracking avian movements in North America

**DOI:** 10.1002/ece3.1383

**Published:** 2015-01-21

**Authors:** Keith A Hobson, Geoff Koehler

**Affiliations:** Environment Canada11 Innovation Blvd., Saskatoon, Saskatchewan, Canada, S7N 3H5

**Keywords:** Assignment, deuterium, isoscapes, oxygen-18, rescaling function, stable isotopes

## Abstract

Tracking migratory animals has benefitted using measurements of naturally occurring stable isotopes of hydrogen (*δ*^2^H) in keratinous tissues such as hair and feathers to link animal origins to continental patterns or isoscapes of *δ*^2^H in precipitation. However, for most taxa, much less information exists on the use of stable oxygen isotope ratios (*δ*^18^O) despite the fact that *δ*^2^H and *δ*^18^O are strongly linked in environmental waters through the meteoric relationship and the possibility of using both isotopes to infer greater information on origins and climatic conditions where tissues are grown. A fundamental requirement of using stable isotopes to assign individuals and populations to origins is the development of a rescaling function linking environmental food web signals to the tissue of interest and for birds, this has not been carried out. Here, we derived the relationship between H and O isotopes in known source feathers of 104 individuals representing 11 species of insectivorous passerines sampled across the strong precipitation isoscape of North America. We determined again a strong expected relationship between feather *δ*^2^H (*δ*^2^H_f_) and long-term amount-weighted precipitation *δ*^2^H (*δ*^2^H_p_; *r*^2^ = 0.77), but the corresponding relationship between *δ*^18^O_f_ and *δ*^18^O_p_ was poor (*r*^2^ = 0.32) for the same samples. This suggests that *δ*^2^H measurements are currently more useful for assignment of insectivorous songbirds to precipitation isoscapes but does not preclude other uses of the *δ*^18^O_f_ data. Currently, mechanisms responsible for the decoupling of H and O isotopes in food webs is poorly known, and we advocate a much broader sampling of both isotopes in the same keratinous tissues across precipitation isotope gradients and across taxa to resolve this issue and to increase the power of using water isotopes to track migratory animals.

## Introduction

**Figure 1:**
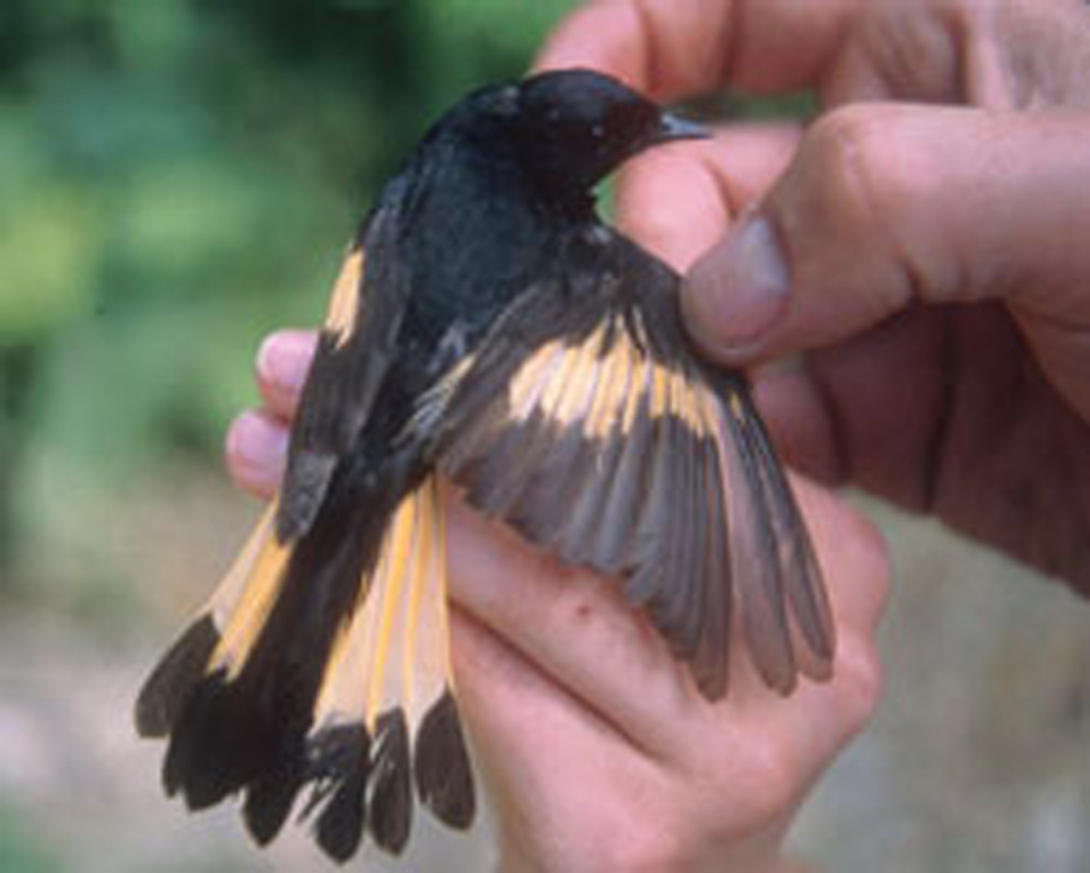
American Redstart (*Setophaga ruticilla*). Feathers sampled from this and other Neotropical migrants have been used to estimate geographical origins using stable isotope analyses.

The measurement of naturally occurring isotopes of the light elements (e.g., C, N, O, H, S) in animal tissues to infer their origins or movements represents a major advance in the study of migration (Fig. 1; Hobson and Wassenaar 2008). In particular, the use of stable hydrogen isotope (*δ*^2^H) measurements now allows inferences of animal origins at continental scales; has led to significant advances in our understanding of movements and origins of birds, insects (Wassenaar and Hobson 1998; Wunder et al. 2005; Brattström et al. 2010; Hobson et al. 2014), bats (Cryan et al. 2014; Voigt et al. 2014), and fish (Soto et al. 2013); and has also been considered as an important forensic tool (Bowen et al. 2005; Hénaux et al. 2011). The isotope approach is based on the fact that (1) food webs incorporate isotopic signals from the environment and pass them on to consumers in a predictable manner typically involving isotopic change or discrimination; (2) such isotopic patterns can become fixed in metabolically inactive tissues such as feathers, claws, and hair and so lock in or archive information; and (3) patterns of isotopes in food webs show spatial structure at local and continental scales (*isoscapes*). Thus, it is possible to associate an animal to a particular isotopic region or isoscape by measuring the stable isotope ratios in its tissues. Central to the application of this method is the establishment of appropriate rescaling functions that link isoscapes to the animal tissue of choice (Bowen et al. 2005, 2014; Wunder 2010; Hobson et al. 2012a). This allows the calibration of an inorganic or food web isoscape to a tissue isoscape that in turn can be used as the basis of the probabilistic spatial assignment of individuals or populations. In the case of hydrogen, animal tissue isoscapes generally follow well-established patterns in long-term amount-weighted precipitation (Hobson 2008). Rescaling functions relating bird feather keratin *δ*^2^H to mean growing season or mean annual precipitation *δ*^2^H have been established for several species and guilds (Cryan et al. 2004; Hobson 2008; Hobson et al. 2012b).

Despite the considerable success in using *δ*^2^H isoscapes to infer origin and movement of animals, there are analytical considerations that complicate the appropriate use of this tool. Hydrogen forms weak bonds with O and N, and this results in a portion of tissue H in proteins exchanging with ambient water vapor. Because laboratory vapor differs in isotopic composition seasonally and across continents, unless this exchangeable portion of H is accounted for, measurements within and between laboratories cannot be readily compared (Meier-Augenstein et al. 2013). Some researchers have countered this problem through the use of appropriate keratin standards of known nonexchangeable *δ*^2^H values (Wassenaar and Hobson 2003), but the field has been plagued by a lack of conformity among laboratories. In contrast, measurements of stable oxygen isotopes (*δ*^18^O) can theoretically overcome this problem because there is a tight relationship between *δ*^2^H and *δ*^18^O in meteoric water (Craig 1961; Dansgaard 1964) and O in tissues does not exchange with ambient water vapor. As with H, there has been little development in the establishment of international standards appropriate for the analysis of *δ*^18^O in animal tissues (Qi et al. 2011), but the routine measurement of *δ*^18^O in animal keratins is now possible. Values of *δ*^18^O in animal tissues occupy a much smaller range than those of *δ*^2^H, but the measurement error is typically smaller (± ∽0.4‰ vs. ± ∽3‰), and so for the purposes of assigning animals to the water-based isoscapes, scale compression should not be a major problem. Sources of H for animal tissues are drinking water and food, whereas those for O include these two sources plus air. However, larger mammals derive proportionally more of their oxygen from drinking water (Bryant and Froelich 1995). While *δ*^18^O measurements have been used extensively in biochemical studies involving animals, these have been restricted primarily to inorganic molecules such as phosphates and carbonates of bones and tooth enamel and insect chitins (reviewed by Pietsch et al. 2011). Recent isotopic analyses of hair have shown a tight relationship between *δ*^2^H and *δ*^18^O for humans (Ehleringer et al. 2008; Thompson et al. 2010) but not for strict carnivores such as felids (Pietsch et al. 2011). So, it remains unknown how effectively *δ*^18^O measurements can be used to assign animals to isoscapes and whether or not the relationship between measured *δ*^2^H and *δ*^18^O in animal tissues contains information that can be related back to environmental factors such as evapotranspiration (Clark and Fritz 1997).

Here, we examined the relationship between the *δ*^18^O and *δ*^2^H values in feathers (*δ*^18^O_f_, *δ*^2^H_f_) with corresponding derived estimates of amount-weighted mean growing season average *δ*^18^O and *δ*^2^H values in precipitation (*δ*^18^O_p_, *δ*^2^H_p_) at feather sampling sites based primarily on the long-term International Atomic Energy Agency (IAEA) Global Network of Isotopes in Precipitation (GNIP; Bowen et al. 2005 and obtained from http://waterisotopes.org). We used feathers grown at known locations across North America, primarily in the United States. We were particularly interested in the relative strength of the two isotopes in reflecting underlying precipitation isoscapes through feathers.

## Materials and Methods

### Samples

**Figure 2:**
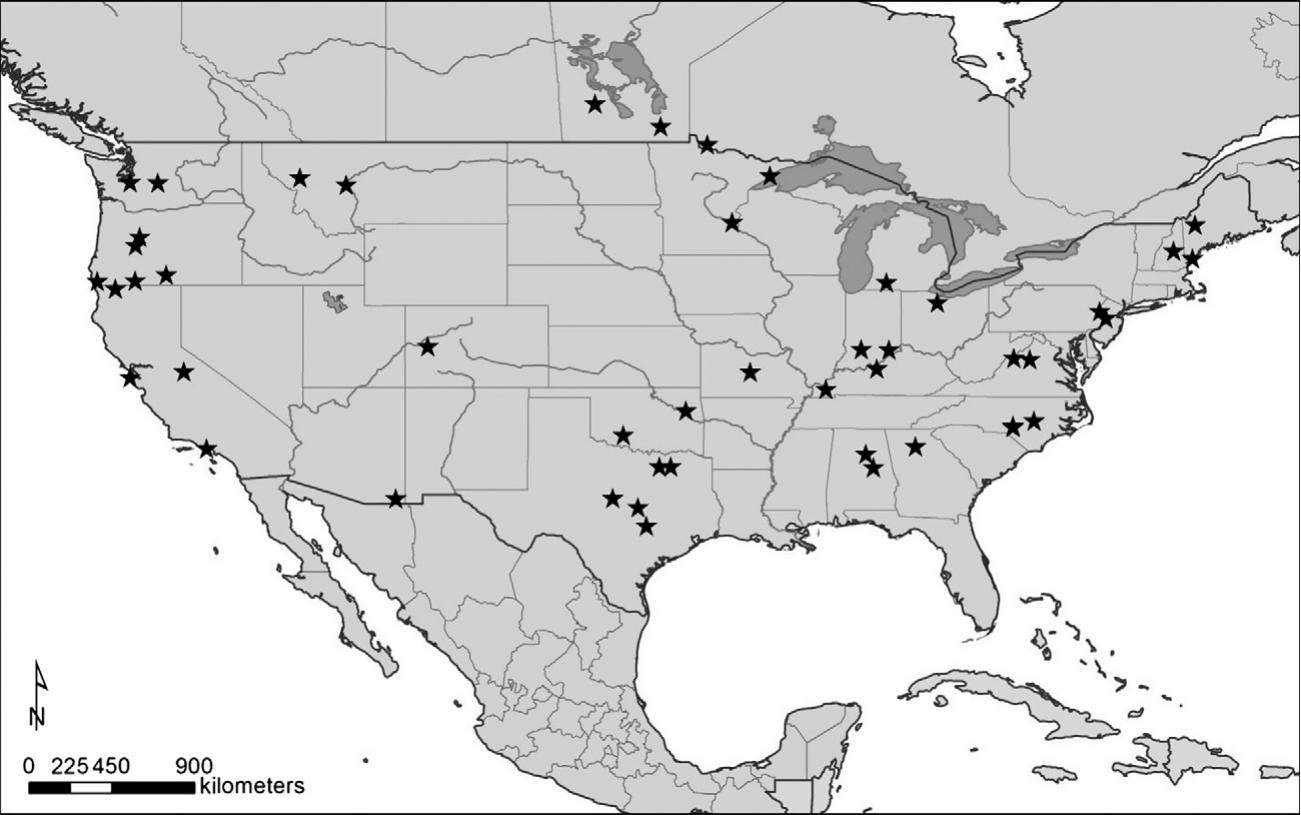
Sampling locations for feathers used in this study. Note that there were 91 unique sampling sites, but only 46 are shown due to local overlap.

The majority (*n* = 96 of 104) of feathers sampled were outer tail feathers (rectrices) of 10 insectivorous passerine species collected during the breeding season at constant-effort mist-netting sites such as MAPS (Monitoring Avian Productivity and Survivorship; DeSante et al. 1995) and by other groups across North America. These samples were selected from a collection stored at the Center for Tropical Research (CTR) at University of California Los Angeles (UCLA) as previously described in Hobson et al. (2012a). Our CTR sample collection was augmented by 8 samples of primary (P1) samples from recaptured Golden-winged Warblers (*Vermivora chrysoptera*) from a mark–recapture experiment. We queried these databases to select only individuals that were captured during the breeding season (May to July) and in at least two different years at the same location. This “recapture” criterion was used to identify individuals that breed at a single location. We further restricted feather sample selection to birds in which the prior capture occurred in the year immediately prior to the sample collection, thereby ensuring that we could be certain of the exact location of feather growth. Birds that were captured during their hatching year and then recaptured in subsequent years were eliminated to avoid confounding age effects. The locations of these sampling sites are shown in Fig. 2. In addition, sampling was restricted to species known to have a complete prebasic (postbreeding) molt on the breeding grounds (Pyle 1997), namely the annual molt of their flight feathers occurring on the breeding grounds, to avoid potential complication of inadvertently sampling a feather grown the previous years. Sample isotope values and collection locations are summarized in Appendix 1.

### Stable isotope analyses

All feathers were cleaned of surface oils in 2:1 chloroform:methanol solvent rinse and prepared for *δ*^2^H and *δ*^18^O analysis at the Stable Isotope Laboratory of Environment Canada, Saskatoon, Canada. Our approach involved the analysis of both *δ*^2^H and *δ*^18^O on the same analytical run (i.e., both H_2_ and CO gases were analyzed from the same pyrolysis) from samples and standards weighed to 350 ± 20 µg in silver capsules. All measurements were taken on a HTC system (Thermo Finnigan, Bremen, Germany) equipped with a Costech Zero-Blank autosampler. The helium carrier gas rate was set to 120 mL/min. We used a new HTC 0.6 m ¼-inch 5-Å molecular sieve (80–100 mesh) GC column. The HTC reactor was operated at a temperature of 1400°C, and the GC column temperature was set to 90°C. After separation, the gases were introduced into a Delta V plus isotope-ratio mass spectrometer via a ConFlo IV interface (Thermo Finnigan, Bremen, Germany). The eluted N_2_ was flushed to waste by withdrawing the CF capillary from the ConFlo interface. We used Environment Canada keratin reference standards CBS (Caribou hoof) and KHS (Kudu horn) to calibrate sample *δ*^2^H (−197 ‰ and −54.1‰, respectively) and *δ*^18^O values (+2.50 ‰ and +21.46 ‰, respectively; Qi et al. 2011). Based on replicate (*n* = 5) within-run measurements of keratin standards, sample measurement error was estimated at ±2 ‰ for *δ*^2^H and ± 0.4 ‰ for *δ*^18^O. All H results are reported for nonexchangeable H and for both H and O in typical delta notation, in units of per mil (‰), and normalized on the Vienna Standard Mean Ocean Water – Standard Light Antarctic Precipitation (VSMOW-SLAP) standard scale.

## Results

**Figure 3:**
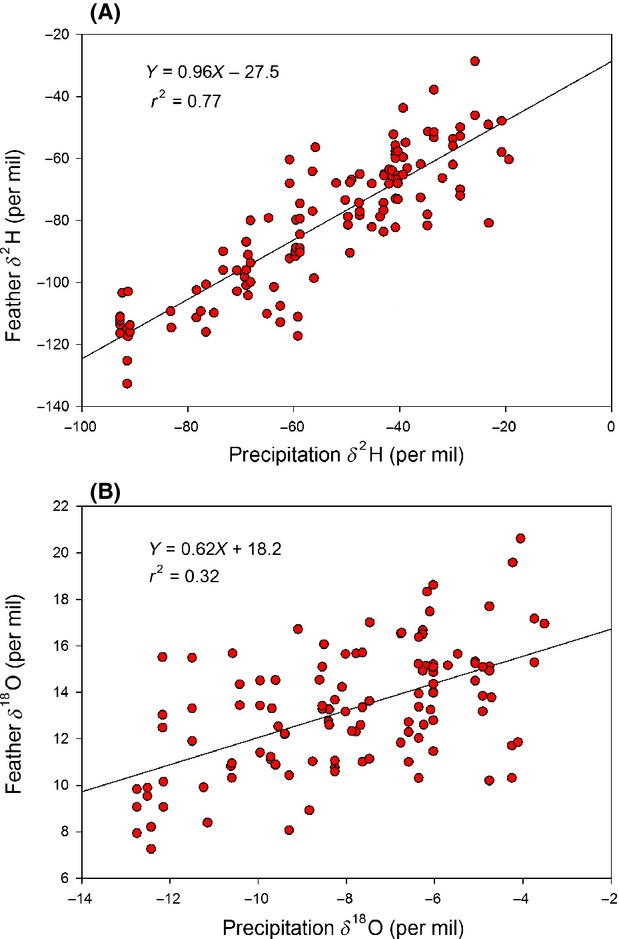
Relationship between (A) feather *δ*^2^H and mean growing season precipitation *δ*^2^H and (B) feather *δ*^18^O and mean growing season precipitation *δ*^18^O predicted for the sampling locations.

**Figure 4:**
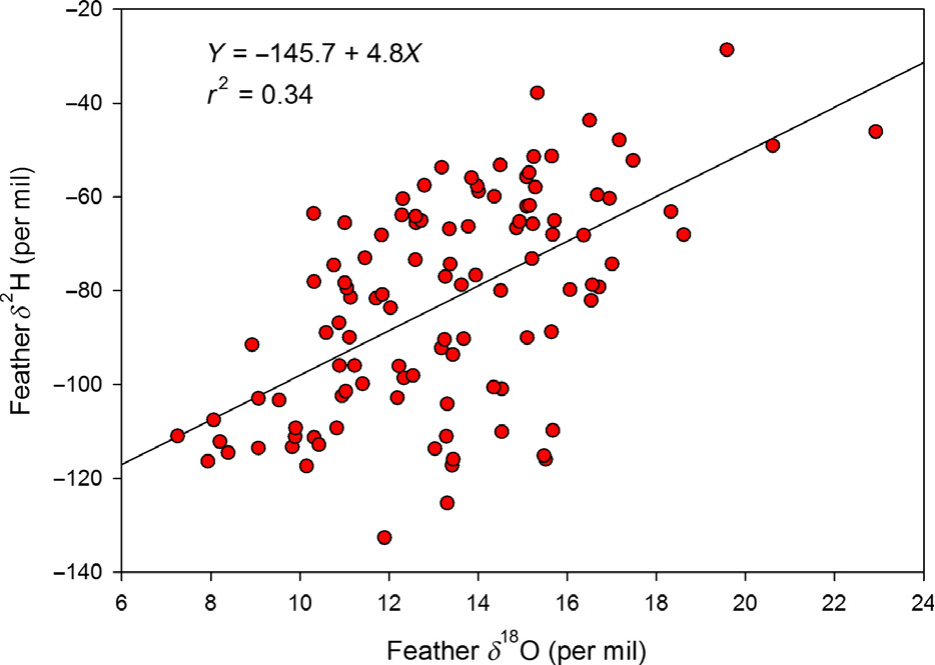
Relationship between feather *δ*^18^O and *δ*^2^H measured for the sample.

As expected, values of *δ*^2^H_p_ and *δ*^18^O_p_ were well correlated for our sites and approached the global mean water relationship (*δ*^2^H_p _= 9.2 + 8.2* *δ*^18^O_p_, *r*^2 ^= 0.98; *P* < 0.001). We found a strong relationship between *δ*^2^H_f_ and *δ*^2^H_p_ (*δ*^2^H_f _= −27.5 + 0.98*δ^2^H_p_, *r*^2 ^= 0.77; *P* < 0.001) but a comparatively weaker relationship between *δ*^18^O_f_ and *δ*^18^O_p_ for the same samples (*δ*^18^O_f _= 18.2 + 0.62*δ^18^O_p_, *r*^2 ^= 0.32; *P* < 0.001; Fig. 3A and B). This resulted in a relatively weak relationship between the two isotopes for feathers (*δ*^2^H_f _= −145.7 + 4.8* *δ*^18^O_f_, *r*^2 ^= 0.34; *P* < 0.001; Fig. 4).

## Discussion

We present the first extensive continentwide test of the relationship between tissue *δ*^18^O and predicted mean growing season precipitation *δ*^18^O for bird feathers in North America. While we confirmed the previously established strong relationship between feather *δ*^2^H and mean growing season precipitation *δ*^2^H (Hobson et al. 2012a) for the same sample, a much weaker relationship was found between feather *δ*^18^O and precipitation *δ*^18^O. This suggests that it will be more challenging to use feather *δ*^18^O isoscapes to assign small insectivorous songbirds to origin due to the much larger inherent variance in the rescaling function linking feather oxygen isotopes to those in precipitation. Our findings also suggest that the breakdown in the meteoric relationship linking *δ*^2^H and *δ*^18^O in food webs may be more related to the behavior of oxygen isotopes versus those of hydrogen (Hobson et al. 2004). This finding has important ramifications for the application of isotopic techniques to tracing animal movements based on keratins but also raises a number of potentially rich research areas that can provide insight into mechanisms influencing isotopic fractionation in vitro and the complexities of animal metabolism.

Previous studies have shown considerable variation in the relationship between O and H isotopes in animal tissues. For birds, Hobson et al. (2009) reported the first relationship between these isotopes in feathers for the American Kestrel (*Falco sparverius*) and found a reasonable linear relationship (*δ*^18^O = 19.36 + 0.09*δ^2^H, *r*^2 ^= 0.48) over a feather *δ*^2^H range of −100 to −20‰ but then no relationship for higher feather *δ*^2^H values. Wolf et al. (2013) conducted a captive study on Japanese Quail (*Coturnix japonica*) raised on different drinking water but found little correlation between feather *δ*^18^O and drinking water *δ*^18^O but a significant correlation between feather *δ*^2^H and drinking water *δ*^2^H (*δ*^2^H_f _= −44.36 + 0.26 *δ*^2^H_w_; *r*^2^ = 0.47). However, that study only tested birds over about a 4.7‰ range in *δ*^18^O drinking water compared with a 53‰ range in drinking water *δ*^2^H (see also Hobson et al. 1999; Wolf et al. 2011). Stronger relationships between O and H isotopes in insect chitin have been reported, especially in the case of aquatic emergents like dragonflies (*r*^2 ^= 0.92, Hobson et al. 2012b) and between chitin and environmental water for brine shrimp (*Artemia franciscana*) raised under controlled conditions (Nielson and Bowen 2010).

Our results contrast with strong relationships found previously between H and O isotopes in environmental waters and human hair (Ehleringer et al. 2008; Bowen et al. 2009; Thompson et al. 2010). Those studies provide support for a mechanistic model that assumed that H and O in ingested proteins undergo exchange with H and O in body waters, which in turn are comprised of H and O from drinking water, food water, and metabolized food. For O, there is a further contribution of O from O_2_ during amino acid breakdown. The model assumes that all H and O atoms within amino acids synthesized in vivo are from body water. H atoms in drinking water comprise the body water pool, and these are available for exchange with amino acids. Thus, the more nonessential amino acids that are biosynthesized in an animal, the more of a H isotope signal derived from drinking water is expected in the animal’s protein pool. For O, a near complete exchange in of carboxyl-bound O during peptide hydrolysis occurs and so O is expected to exchange with body water for both essential and nonessential amino acids resulting in O isotope ratios in keratins being insensitive to the extent of in vivo synthesis (Thompson et al. 2010). The relative influence of H and O isotopes in environmental waters to those in keratins is expected to be affected by the extent of in vivo amino acid synthesis with more of an influence on H in cases of higher levels of synthesis.

In addition to the differences among animals in their use of essential versus nonessential amino acids, animals vary in their relative use of drinking water versus metabolic water as a contribution to the body water pool available for H and O isotopic exchange. Pietsch et al. (2011) proposed that the breakdown in the transference of an environmental water isotope signal to wild (Felid) carnivores was due to the minor role of drinking water contribution to the body water pool compared to that in herbivores or more well-hydrated carnivores. Similarly, Bowen et al. (2009) found that mid-20th Century Inuit with a highly carnivorous (marine) diet showed a poorer relationship between hair O and H isotopes and meteoric drinking water. Relative humidity has been shown to affect *δ*^18^O values in bone phosphate and *δ*^2^H in bone collagen of herbivores (Ayliffe and Chivers 1990; Cormie et al. 1994), but Pietsch et al. found inconsistent effects of RH on hair *δ*^18^O values in felids.

The addition of *δ*^18^O measurements to *δ*^2^H measurements in keratinous tissues of migratory animals has the potential to provide important additional information on source environmental waters, diets, and climatic conditions during and prior to growth. However, it is currently not well understood which mechanisms contribute to a poorer meteoric signal for *δ*^18^O in some animals compared with *δ*^2^H and under what circumstances such information can be gleaned. With the advent of useful protocols for the routine measurement of *δ*^2^H and *δ*^18^O in complex organics (Qi et al. 2011; Meier-Augenstein et al. 2013), there is now considerable potential for future research in this area that will include the survey of patterns across taxa and under controlled conditions.

## Appendix 1

Summary information for locations of the feather samples used and results of *δ*^2^H and *δ*^18^O analyses. Species are identified as four-letter codes: AMRE, American Redstart (*Setophaga ruticilla*); BAWW, Black-and-white Warbler, (*Mniotilta varia*); CACH, Carolina Chickadee (*Poecile carolinensis*); CARW, Carolina Wren (*Thryothorus ludovicianus*); COYE, Common Yellowthroat (*Geothlypis trichas*); ETTI, Eastern Tufted Titmouse (*Baeolophus bicolor*); GWWA, Golden-winged Warbler (*Vermivora chrysoptera*); HOWA, Hooded Warbler, (*Wilsonia citrina*); MGWA, McGillivray’s Warbler (*Oporornis tolmiei*); SOSP, Song Sparrow (*Melospiza melodia*); WEVI, White-eyed Vireo (*Vireo griseus*); WIWA, Wilson’s Warbler, (*Wilsonia pusilla*); YBCH, Yellow-breasted Chat (*Icteria virens*); YWAR, Yellow Warbler, (*Setophaga petechia*).

**Table:**

Species	Year	Latitude	Longitude	*δ*^2^H (‰)	*δ*^18^O (‰)
AMRE	1999	38.40556	−78.4972	−67	13.4
AMRE	1999	38.55833	−78.3778	−65	15.7
AMRE	1999	38.55833	−78.3778	−78	11.0
AMRE	2004	44.98278	−70.4164	−108	8.1
AMRE	2004	37.7425	−92.0428	−58	12.8
AMRE	2007	47.38222	−91.1964	−87	10.9
AMRE	2007	47.38222	−91.1964	−96	10.9
AMRE	2007	47.38222	−91.1964	−101	14.5
AMRE	2007	37.7425	−92.0428	−73	11.5
AMRE	2007	41.18056	−83.0222	−79	16.6
BAWW	2007	35.8883	−95.3066	−53	14.5
CACH	2006	31.6497	−98.9051	−29	19.6
CACH	2007	31.6176	−98.8898	−46	22.9
CACH	2007	35.8883	−95.3066	−51	15.2
CARW	2006	30.2591	−97.258	−60	16.9
CARW	2007	34.6901	−98.3691	−54	13.2
CARW	2007	34.6901	−98.3691	−56	13.8
CARW	2007	35.8883	−95.3066	−38	15.3
CARW	2006	38.88556	−86.7367	−77	13.9
CARW	2007	40.41111	−74.775	−73	12.6
COYE	1999	37.81194	−85.8281	−65	12.6
COYE	1999	43.33611	−70.5472	−77	13.3
COYE	1999	43.33611	−70.5472	−64	12.6
COYE	1999	35.13889	−79.3278	−55	15.1
COYE	1999	44.37083	−122.016	−90	11.1
COYE	2000	38.86	−85.38	−65	12.7
COYE	2000	44.37083	−122.016	−96	11.2
COYE	2001	38.88556	−86.7367	−84	12.0
COYE	2001	40.73889	−75.0917	−79	13.6
COYE	2001	40.73889	−75.0917	−81	11.1
COYE	2004	44.98278	−70.4164	−113	10.4
COYE	2006	38.88556	−86.7367	−74	13.4
COYE	2006	43.69167	−71.475	−92	8.9
COYE	2007	39.04639	−85.4394	−66	15.2
COYE	2007	42.16667	−85.5194	−68	11.8
COYE	2007	45.09722	−93.0633	−75	10.8
COYE	2007	45.09722	−93.0633	−89	10.6
COYE	2007	45.09722	−93.0633	−79	11.1
ETTI	2007	34.6901	−98.3691	−62	15.1
ETTI	2007	38.455	−79.2933	−74	17.0
GWWA	2008	50.9991	−100.065	−113	9.8
GWWA	2008	50.99008	−100.067	−114	9.1
GWWA	2009	49.78144	−96.5539	−109	9.9
GWWA	2009	48.85506	−94.6224	−109	10.8
GWWA	2009	48.89714	−94.2585	−111	10.3
GWWA	2009	49.64905	−96.2417	−115	8.4
GWWA	2009	50.99538	−100.069	−116	7.9
GWWA	2009	50.78194	−99.6101	−117	10.1
GWWA	2009	51.02679	−100.036	−112	8.2
GWWA	2009	51.00099	−100.069	−111	7.3
HOWA	2001	33.11667	−86.1389	−51	15.7
HOWA	2004	38.80861	−86.8814	−64	10.3
HOWA	2006	38.80861	−86.8814	−68	16.4
MGWA	1996	39.04063	−107.942	−110	14.5
MGWA	1996	41.84333	−123.21	−79	16.7
MGWA	1996	42.3925	−124.119	−92	13.2
MGWA	1996	42.6425	−124.119	−89	15.6
MGWA	1996	42.22778	−124.097	−68	15.7
MGWA	1996	42.22778	−124.097	−60	12.3
MGWA	1996	42.60833	−123.853	−80	16.1
MGWA	1996	43.88611	−122.264	−94	13.4
MGWA	1996	46.80556	−121.053	−110	15.7
MGWA	2005	42.24806	−122.234	−103	12.2
MGWA	2007	37.79472	−119.864	−90	15.1
MGWA	2007	42.24806	−122.234	−96	12.2
MGWA	2007	42.52	−120.711	−104	13.3
MGWA	2007	43.88194	−122.2	−100	11.4
MGWA	2007	42.67306	−120.816	−98	12.5
MGWA	2007	47.02194	−121.138	−101	14.3
SOSP	2002	34.03778	−118.746	−82	11.7
SOSP	2002	34.03778	−118.746	−78	10.3
WEVI	2003	34.13787	−84.0921	−63	18.3
WEVI	2005	33.15748	−96.6153	−49	20.6
WEVI	2006	31.1638	−97.6358	−48	17.2
WEVI	2003	35.39861	−78.2894	−60	16.7
WEVI	2003	35.39861	−78.2894	−65	14.9
WEVI	2006	33.15167	−96.05	−81	11.8
WEVI	2007	36.92778	−88.4642	−52	17.5
WEVI	2007	37.69444	−92.1111	−56	15.1
WEVI	2007	37.77389	−92.2008	−58	14.0
WEVI	2007	37.705	−92.1167	−60	14.4
WEVI	2007	35.39861	−78.2894	−44	16.5
WEVI	2007	31.15389	−97.6658	−58	15.3
WIWA	1996	37.50556	−122.497	−66	13.8
WIWA	2002	47.05556	−122.488	−101	11.0
WIWA	2003	43.95806	−122.213	−80	14.5
YBCH	2001	38.96639	−85.4583	−64	12.3
YBCH	2006	33.76194	−86.5189	−62	15.2
YBCH	2006	38.86	−85.38	−65	11.0
YBCH	2006	37.69444	−92.1111	−59	14.0
YBCH	2007	37.77889	−92.1797	−73	15.2
YBCH	2007	37.77889	−92.1797	−67	14.9
YBCH	2007	37.77889	−92.1797	−68	18.6
YBCH	2007	42.49028	−123.48	−99	12.3
YWAR	2007	31.5876	−109.505	−90	13.2
YWAR	2000	37.70444	−119.752	−117	13.4
YWAR	2000	46.93361	−111.915	−116	15.5
YWAR	2001	37.70444	−119.752	−111	13.3
YWAR	2002	42.16667	−85.5194	−82	16.5
YWAR	2002	46.93361	−111.915	−114	13.0
YWAR	2003	47.27371	−114.178	−115	15.5
YWAR	2003	47.27371	−114.178	−125	13.3
YWAR	2003	47.27371	−114.178	−133	11.9
YWAR	2004	47.02194	−121.138	−116	13.4
YWAR	2007	45.09722	−93.0633	−90	13.7
